# Revisiting the Role of Neurotrophic Factors in Inflammation

**DOI:** 10.3390/cells9040865

**Published:** 2020-04-02

**Authors:** Lucas Morel, Olivia Domingues, Jacques Zimmer, Tatiana Michel

**Affiliations:** Department of Infection and Immunity, Luxembourg Institute of Health, 29 rue Henri Koch, L-4354 Esch-sur Alzette, Luxembourg; lucas.morel@lih.lu (L.M.); olivia.domingues@lih.lu (O.D.); jacques.zimmer@lih.lu (J.Z.)

**Keywords:** GDNF family ligands (GFLs), inflammation, epithelial-neuronal signaling, immune cells

## Abstract

The neurotrophic factors are well known for their implication in the growth and the survival of the central, sensory, enteric and parasympathetic nervous systems. Due to these properties, neurturin (NRTN) and Glial cell-derived neurotrophic factor (GDNF), which belong to the GDNF family ligands (GFLs), have been assessed in clinical trials as a treatment for neurodegenerative diseases like Parkinson’s disease. In addition, studies in favor of a functional role for GFLs outside the nervous system are accumulating. Thus, GFLs are present in several peripheral tissues, including digestive, respiratory, hematopoietic and urogenital systems, heart, blood, muscles and skin. More precisely, recent data have highlighted that different types of immune and epithelial cells (macrophages, T cells, such as, for example, mucosal-associated invariant T (MAIT) cells, innate lymphoid cells (ILC) 3, dendritic cells, mast cells, monocytes, bronchial epithelial cells, keratinocytes) have the capacity to release GFLs and express their receptors, leading to the participation in the repair of epithelial barrier damage after inflammation. Some of these mechanisms pass on to ILCs to produce cytokines (such as IL-22) that can impact gut microbiota. In addition, there are indications that NRTN could be used in the treatment of inflammatory airway diseases and it prevents the development of hyperglycemia in the diabetic rat model. On the other hand, it is suspected that the dysregulation of GFLs produces oncogenic effects. This review proposes the discussion of the biological understanding and the potential new opportunities of the GFLs, in the perspective of developing new treatments within a broad range of human diseases.

## 1. Introduction

The glial cell line-derived neurotrophic factor (GDNF) family of ligands (GFLs), comprising GDNF, neurturin (NRTN), artemin (ARTN) and persephin (PSPN), are proteins structurally related to transforming growth factor (TGF)-β and signal through the glycosyl-phosphatidylinositol (GPI)-anchored coreceptors (GFRα1–4), as a ligand binding component, and Ret receptor tyrosine kinase as a signaling component [1]. GDNF predominantly binds to GFRα1, NRTN to GFRα2, ARTN to GFRα3, and PSPN to GFRα4. Various signaling pathways are activated, such as RAS/mitogen activated protein kinase (MAPK), RAS/extracellular signal-regulated kinase (ERK), phospholipase C gamma (PLCγ), phosphatidylinositol-3-kinase (PI3K)/protein kinase B (AKT) and c-Jun *N*-terminal kinase (JNK) pathways, which contribute to cell adhesion, migration, proliferation, differentiation and survival. However, GFRα may also signal independently of Ret via the neural cell adhesion molecule (NCAM) pathway [2]. In addition, heparan sulfate proteo-glycosaminoglycans (HSPG) can serve as co-receptors of Ret to transduce GFLs signals, leading to downstream activation via Src kinases [3]. This family is well described for its role in the growth and the maintenance of the peripheral and central nervous systems and acts on dopaminergic, sensory, motor, hippocampal, midbrain, enteric, sympathetic and parasympathetic neurons [4]. As GFLs are able to control multiple processes in neuronal cells, including neurite outgrowth, neuronal plasticity, formation of synapses, migration and survival, they have been assessed in clinical trials as a treatment for neurodegenerative diseases, such as Parkinson’s disease [5,6,7]. Moreover, GDNF is largely studied in neuromuscular pathologies, as it can prevent motor neuron degeneration in animal models of amyotrophic lateral sclerosis (ALS) and it represents a possible biomarker to predict ALS development [8,9]. Recently, the delivery of recombinant adeno-associated virus serotype-2 (rAAV2)-NRTN in ALS mice has shown a neuroprotective capacity in slowing disease progression [10].

Although GFLs are defined as molecules that maintain neuronal cells, they possess a range of functions outside the nervous system. During embryogenesis, the GDNF, NRTN, GFRα1, and GFRα2 mRNA expression patterns have been identified, most notably in epithelial-mesenchymal interactions, such as during skin, kidney, stomach, testis, lung, and tooth development. Thus, they play a critical role in the outgrowth of epithelial structures [11,12]. At last, GFLs are also involved in the regulation of hematopoietic cell differentiation [13] and in the activation of the immune system response during infections. In this review, we will highlight the functions of GFLs and their emerging role in inflammation in different diseases, with the perspective of the development of new treatments.

## 2. Phenotype Associated with GFLs and Their Receptor Defects

Within the different knockout (^−/−^) mice for GFLs members and receptors, the GDNF, GFRα1 and Ret null mutant mice die at birth and have phenotypic similarities, including defects in enteric nervous system, kidney and sensory neurons [14,15,16]. NRTN^−/−^ and GFRα2^−/−^ mice have smaller parasympathetic ganglia and multiple defects of a variety of peripheral neurons and in epithelial function, due to reduced innervation of the gastrointestinal, lacrimal, salivary and testicular glands [17,18,19,20]. In addition, the secretion of inflammatory cytokines is dramatically increased in NRTN^−/−^ mice [21]. Indeed, it has been observed that the expression of IL-1β, TNF-α, and matrix metalloproteinase (MMP)-9 was upregulated by the corneal epithelia in NRTN^−/−^ mice compared to WT mice. On another side, the GFRα3, GFRα4, ARTN and PSPN deficient mice don’t show major phenotypic impairments [22,23], only ARTN^−/−^ and GFRα3^−/−^ mice present abnormalities in the sympathetic nervous system. Thus, GFLs members seem to have different roles to play during the development and the adult stage of the mice. This review presents the different aspects of their activities.

## 3. Neurotrophic Factors in Inflammation

**Figure 1:**
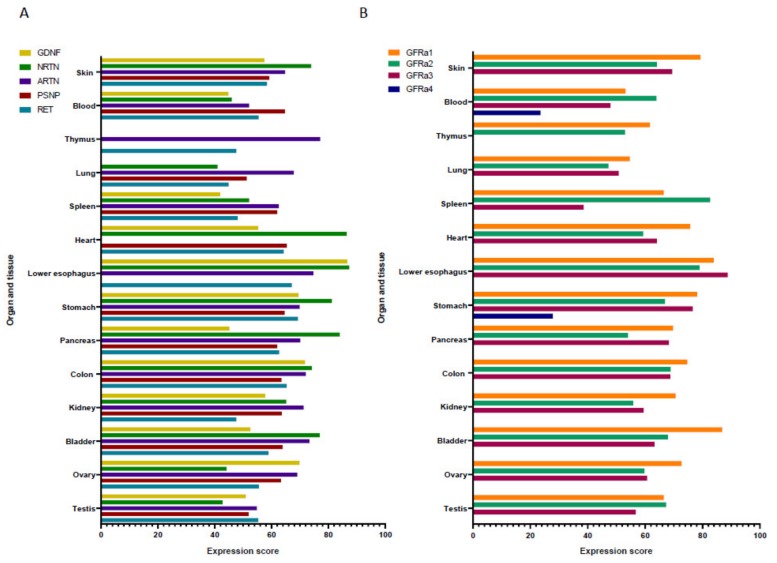
Expression score of the GDNF family ligands (GFLs) and their receptors in a non-exhaustive list of organs and tissues. Data of GFLs (**A**) and their receptors (**B**) are from the Bgee database (https://bgee.org/). They have been obtained by RNAseq and affimetrix microarray from 1673 healthy donors in total, aged between 45 and 65 years old. The expression score from adult healthy donors has been normalized to a value between 0 and 100 from rank scores. (Bgee version 14.0).

GFLs are expressed during inflammation outside the nervous system in several peripheral tissues in both human and mouse [12], suggesting that their physiological role is not solely confined to the nervous system (Figure 1). In addition, the expression and the regulation of GFLs are modulated during inflammation, in the presence of bacteria, lipopolysaccharides (LPS) and inflammatory cytokines, such as TNF-α, IL-6 and IL-1β [24,25,26,27].

### 3.1. Interaction and Expression in Immune Cells

Recent studies have described that GFLs have a link with immune cells. GFLs are expressed in several human tissues linked to the development and function of immune cells, like the thymus, lungs and spleen (Figure 1). GDNFRβ (today known as GFRα2) and NRTN expression have been found in the spleen and lung tissues of rats [28]. Moreover, immunohistochemistry data show that GFRα3 is expressed in the human spleen [29]. In the thymus, which is a specialized primary lymphoid organ of the immune system, Ret is present in cells with a dendritic aspect in rat, whereas in human, Ret is predominantly expressed by thymic epithelial cells [29]. In addition, GFRα1, together with GDNF, were found in the thymus of adult mice [30]. GFRα1 is present in immature thymocytes and its expression decreases with the maturation of the cells. On the other hand, Gattei and al. have described that Ret is expressed in low amounts in human hematopoietic progenitor cells, but the expression rate increases with cell maturation [13,31]. In mice, GFLs present in bone marrow hematopoietic stem cells (HSC) environment activate the Ret signaling in HSCs, which improves cell survival, expansion and function in transplantation efficiency [32].

**Figure 2:**
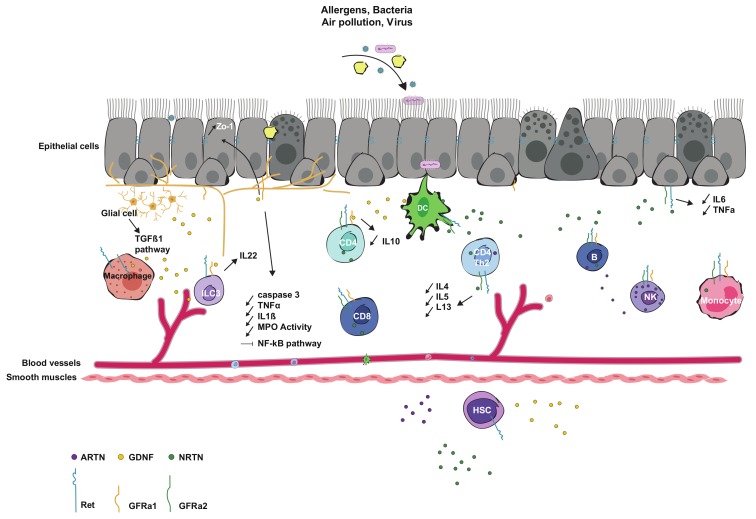
Overview of the anti-inflammatory roles of GFLs and their receptors following air pollution, allergy, bacteria or virus infections. Depending on the type of inflammation, the release of GFLs by epithelial, glial or dendritic cells will lead to the activation of immune cells expressing GFRα and Ret, located in blood and near gut, corneal, skin and lung epitheliums. The consequences of the GFLs stimulation in the gut will lead to the secretion of IL-22 by ILC3, the activation of the TGF-β1 pathway, the upregulation of the tight junction proteins such as ZO-1 in epithelial cells and the downregulation of caspase 3, the pro-inflammatory cytokines TNF-α and IL-1β and the MPO. In addition to the reduction of inflammatory cytokine secretion, GDNF contributes to the inhibition of the NF-κB signaling pathway by the corneal epithelial cells. In the lung environment, a decrease of IL-10 expressed by T cells or less release of IL-6 and TNF-α by the epithelial cells are observed. After allergic airway inflammation, the presence of NRTN will dampen the reaction through the downregulation of the Th2 cytokines. MPO: myeloperoxidase; DC: dendritic cells; B: B lymphocytes; NK: natural killer cells; ILC: innate lymphoid cells; HSC: hematopoietic stem cells; CD4: CD4^+^ helper T lymphocyte; CD8: CD8^+^ cytotoxic T lymphocyte.

Ret, NRTN, GFRα1 and GFRα2 are expressed on both innate (monocytes and Natural Killer cells) and adaptive (B lymphocytes, CD4^+^ and CD8^+^ T cells) human peripheral blood mononuclear cells (PBMCs), suggesting a putative role of the GFLs in modulating immune cell responses [33,34,35,36,37]. In addition, RET seems to contribute to the development and maintenance of the immune system, as its expression is increasing in differentiated monocytes, like macrophages [37]. Moreover, several inflammatory genes, such as those encoding chemokines (CCL20, CCL2, CCL3, CCL4, CCL7, CXCL1) and cytokines (IL-1β, IL-6 and IL-8) are upregulated by RET in PBMCs, especially in monocytes/macrophages from healthy donors after treatment with GDNF and GFRα1 [35]. The GFL family co-receptor is also involved in the regulation of IL-10 production in Th2 cells, as the absence of Ret leads to an increased production of this cytokine, while the presence of GDNF and NRTN downregulates its expression [36]. In addition, ARTN is also expressed in T cells, B cells and NK cells, but the expression level is lower than for the other GFL members [37]. Finally, it has been described that Ret is expressed by enteric innate lymphoid cells (ILC)3 when they are in proximity to glial cells, helping the regulation of innate IL-22 production [38] and that Ret with GFRα3 are expressed in the hematopoietic cells involved in Peyer’s patch formation [39]. GDNF is also secreted by macrophages and upregulated in the presence of LPS [40] and its expression is detected in eosinophils from the duodenum of patients with functional dyspepsia [41]. In the context of brain immune cells, GDNF and its receptors are expressed in microglia and related macrophages in inflammatory conditions, such as striatal injury [42,43], spinal cord transection [27] or LPS induction [44,45,46]. Thus, from hematopoietic lymphoid tissue initiators to adult immune cells, GFLs are expressed and upregulated in the context of inflammation, suggesting a putative function to modulate the immune cell response (Figure 2). However, further studies are needed to determine the exact role of these GFLs during inflammation.

### 3.2. Interaction and Expression in Epithelial Cells

It has been described that GDNF is participating in the regulation of microvascular endothelial and epithelial tight junction barrier function in mammalian blood-brain, blood-retina, blood-testis and intestinal barriers, in vitro and in vivo.

In intestinal barriers, enteric glia cells (EGC) regulate and protect intestinal epithelial cell invasion from bacterial infection and their protective role is partly mediated through the activation of the TGF-β1 pathway [47,48,49]. Following injury or infection, EGC release GDNF, which contributes to the protection of the intestinal epithelial cells, by upregulating the expression of the tight junction proteins. Thus, GDNF protects the gut epithelium by mediating the upregulation of the expression of ZO-1 and the inhibition of the mucosal inflammatory response, through the downregulation of caspase 3, the pro-inflammatory cytokines TNF-α and IL-1β and the myeloperoxidase (MPO) activity [50,51,52,53]. GDNF also presents anti-inflammatory properties in the human corneal epithelium, as it has been reported that GDNF enhances the corneal epithelial cell survival, proliferation, migration and immunoprotection via GFRα1 [54]. In addition, GDNF suppresses the production of IL-17-induced inflammatory cytokines, such as TNF-α, IL-1β, IL-6 and IL-8, by blocking the NF-κB signaling pathway on primary human corneal epithelial cells [55].

NRTN is expressed at high levels in the epithelium and mucosa of the female reproductive tract in the oviduct and within the ovary, suggesting that NRTN may be involved in the process of follicle maturation or ovulation [56]. NRTN is also detected in human serum and in case of premature ovarian insufficiency, NRTN is downregulated in the serum of the patients compared to the healthy fertile and menopausal groups. Thus, NRTN may be a novel serum biomarker in patients with premature ovarian insufficiency [57].

In skin barrier, the expression of ARTN is upregulated in part by keratinocytes, in response to inflammation induced by hind paw injection of complete Freund’s adjuvant in mouse and rat models [58,59]. The elevated level of ARTN in deep layers of the epidermis and over a broad area of the dermis after inflammation, leads to the induction of hyperalgesia through the sensitivity of thermal nociceptors. In addition to ARTN, the expression level of GDNF produced by keratinocytes in the skin also increases during inflammatory hyperalgesia and participates to the induction of the neuronal transient receptor potential ion channel, TRPV1 pathway [60]. Thus, during peripheral inflammation, ARTN and GDNF elicit thermal hyperalgesia, and maintaining normal levels of these GFLs might be an effective therapeutic approach to treat this inflammatory pain.

NRTN, which is produced by the salivary gland epithelium during development, participates in the maintenance of neuronal-epithelial interactions in cases of damage, such as therapeutic irradiation (IR) in cancer. Indeed, NRTN restores parasympathetic innervation affected by IR and increases salivary gland epithelial regeneration via GFRα2 and the upregulation of the cell adhesion molecule E-cadherin [61]. Thus, NRTN is a good candidate to improve epithelial organ regeneration. It has been shown that PSPN also has epithelial cell protective capacities. In experiments on cell lines, the release of PSPN by the perivascular cells participates to increase the proliferation of the lung alveolar epithelial cells, which express GFRα4 [62]. In addition, the presence of the Th2 type cytokine IL-13 induces the expression of NRTN by the airway epithelial cells [63]. In a mouse model of allergic airway inflammation, NRTN participates to maintain the airway epithelium integrity and decreases the level of inflammation [64,65]. The GFLs are key players during inflammation, for the communication between neuronal and epithelial cells (Figure 2), and a better comprehension of the signaling pathways controlled by GFLs in the epithelia could lead to their use as new targets for therapeutic development.

### 3.3. Interaction with Microbiota

More and more data show the influence of the commensal gut microbiota in the development and progress of a variety of enteric, metabolic and inflammatory disorders. The absence of gut microbiota in germ-free mice induces an upregulation of the expression of GDNF in the hippocampus of these mice compared to specific-pathogen-free mice, which could lead to the reduced anxiety-like behaviors in the former [66]. However, further studies on the exact role of the GDNF pathway on neuropsychiatric disorders need to be performed. On the other hand, wild-type mice depleted of intestinal microbiota had enteric nervous system (ENS) defects associated with a decreased level of GDNF in the longitudinal smooth muscle-myenteric plexus [67]. Another study described that following inflammation, gut microbial components like pathogen-associated molecular patterns are able to enhance the production of GDNF by the intestinal smooth muscle cells via TLR2 engagement [68]. The secretion of GDNF leads to preservation of the enteric neuron integrity, which points out an active role of GDNF in the regulation of the intestinal contractility affecting smooth muscle cells. In addition, the protection of the intestinal tract against microbial inflammation through interactions between EGCs and the immune cells ILC3 via GFL signaling has recently been reported. ILC3, which expresses Ret, induce the release of IL-22 after activation by GDNF derived from EGCs [38]. Thus, GFLs participate in intestinal repair as well as promote efficient gut homeostasis and defense via the regulation of IL-22.

## 4. Roles of GFLs in Pathologies

### 4.1. In Gut Diseases

Patients with Hirschsprung’s disease suffer from partial to complete intestinal obstruction, due to variable lengths of aganglionosis in the hindgut. This disease is often caused by loss-of-function mutations in Ret affecting the tyrosine kinase domain [69,70]. The Hirschsprung-associated Ret mutants show a constitutive pro-apoptotic activity, which could be the direct cause of the disappearance of the neural crest-derived cells in the hindgut [71]. In mouse, the reduction of 80% of GFRα1 expression leads to the Hirschsprung’s disease phenotype found in untreated children [72]. In Crohn’s disease, the EGC are a major cellular source of the upregulated GDNF in the inflamed gut. In case of colitis, GDNF has the capacity to regulate the intestinal barrier function and inflammation by reducing epithelial cell apoptosis [51].

### 4.2. In Kidney Diseases

The administration of adipose-derived mesenchymal stem cells expressing GDNF exerts an anti-inflammatory mechanism in the renal interstitial fibrosis mouse model, presenting a potential new therapy in the case of kidney pathologies. This effect is mediated by the regulation of macrophage activity, through the downmodulation of the inflammation-related proteins IL-6 and COX-2 and the expression levels of the M2-related cytokines IL-4 and IL-10 [73]. In another context, as the GDNF level is upregulated in the urine of interstitial cystitis patients, it could serve as a marker for monitoring inflammation [74].

### 4.3. In Diabetes

As NRTN was identified as a secreted factor in embryonic pancreas, it was used to prevent or reverse the progression of diabetes. The injection of NRTN in a diabetic rat model prevents the development of hyperglycemia, and reduces fasting glucose levels and the deterioration of metabolic parameters. However, it is not clear how NRTN mediates its metabolic activity to prevent hyperglycemia [75]. Diabetes can also result in the loss of enteric neurons leading to gastrointestinal dysfunction. Hyperglycemia is associated with enteric neuronal apoptosis, but this phenotype can be reverted by GDNF in diabetic mice via the activation of the PI3K/Akt signaling [76]. In another context, GDNF and GFRα1, which are expressed by Müller cells in the retina, have a protective role in a high glucose environment in the case of diabetic retinopathy. GDNF treatment ameliorates diabetes associated with Müller cells impairment, but also promotes the synthesis of endogenous GDNF and its receptors [77].

### 4.4. In Asthma and Allergic Rhinitis

Several studies have described links between GFLs and airway inflammation. First, data from guinea pig reveal that GFL members may be important for the neuromodulation of vagal nerves in the adult respiratory tract, as GFRα1, GFRα2, GFRα3 and Ret are expressed in the majority of these neurons. In addition, the expression of GDNF and NRTN has been shown in the airway smooth muscle cells [78], and GFRα2, Ret and NCAM were found especially in respiratory epithelial cells present in the bronchus and in the nasopharynx, but also in macrophages [79]. During allergic airway inflammation, a production of GDNF is observed in the airway mucosa, as well as in the tracheal epithelium [80]. Our group has analyzed the impact of an allergic airway inflammation induced by two different allergens, ovalbumin and house dust mite, in NRTN*^−/−^* mice. The absence of NRTN correlates with an increased number of eosinophils and Th2 responses in lung tissue and lung draining lymph node cells [64,65]. Following inflammation, NRTN*^−^*^/*−*^ mice display increased markers of airway remodeling like collagen deposition, higher levels of neutrophils, matrix metalloproteinase (MMP-9), TNF-α and IL-6, and treatment with NRTN partially rescued the phenotype observed [64,65]. At the human level, it has been shown that GFRα2 and NCAM are expressed in nasal polyps and are downregulated in patients with eosinophilic chronic rhinosinusitis [81]. Additionally, the analysis of nasal samples from grass-pollen allergic patients receiving allergen-specific immunotherapy shows the downregulation of the expression of GFLs and their receptors versus untreated patients [82]. Finally, in another context, the infection with the bacterium *Streptococcus pneumoniae*, causing diseases such as pneumonia, sinusitis and meningitis, alters the expression level of GDNF in the olfactory bulb, which could indirectly influence microglial activation [83].

### 4.5. In Skin Diseases

GFLs are associated with itchining, one of the symptoms of psoriasis. An increase of nonpeptidergic epidermal nerve density and skin NRTN expression is characteristic for this disease. Blocking the NRTN/GFRα2 pathway in an induced-psoriasis mouse model leads to a reduction of the nonpeptidergic nerve density as well as spontaneous scratching, a behavior associated with chronic itch [84]. Additionally, in the context of atopic dermatitis, where intense chronic itch is also observed, ARTN is released in the epidermis of patients via the activation of the aryl hydrocarbon receptor (AhR) by particulate matter in air pollution [85,86]. The accumulation of ARTN in human dermal fibroblasts from atopic dermatitis lesions induces epidermal hyper-innervation. This effect could be responsible for the scratching behavior, as the injection of ARTN in mice causes rubbing which mimics scratching itch [87]. Targeting ARTN signaling could be a new way to treat atopic dermatitis patients with aggravated symptoms, due to environmental pollutants, for whom steroids are ineffective.

### 4.6. In Neuropsychiatric Disorders

Altered levels of GDNF were observed in association with depression and the analysis of the blood of mood disorder patients has shown a reduction of GDNF and ARTN during the depressive state [88]. Thus, dysfunctions of GFRα1-mediated pathways may contribute to oxidative stress-induced cell death and apoptosis in peripheral blood cells of mood disorder patients. This might be one of the mechanisms of immune inadequacy in the pathophysiology of major depression. Another aspect of the regulation of the hematopoetic cells by the GFLs, is the capacity to maintain the bone marrow homeostasis by controlling hematopoetic stem cells and leukocyte traffic. Thus, the regulation of hematopoetic stem cell egress from bone marrow to circulation is induced by the cholinergic signaling via the NRTN/GFRα2 pathway [89].

### 4.7. In Cancers

Exposure to a variety of toxic compounds, such as alcohol or nicotine, is associated with increased cancer risk, stimulates angiogenesis and epithelial to mesenchymal transition (EMT). This step is critical in cancer progression and can induce plasticity in multiple tissues, including nerves. Cancer cells can communicate with the peripheral nervous system, through the release of molecules that are also made by neurons, including the GFLs, ARTN, GDNF, and NRTN [90]. Tumors, via the expression of GFRα1, GFRα2, GFRα3, respond to molecules released by nerves and, in an autocrine fashion, to molecules released by the tumor itself and these interactions are likely amplified in the tumor microenvironment. Thus, GFLs are upregulated in a variety of cancer cells of epithelial origin: pancreatic, testicular, bile duct, colon, mammary, endometrium, ovarian and lung, as well as glioma, and their increased expression is associated with malignant progression and a poor prognosis [91]. ARTN and GDNF are the two major GFLs participating to the reduction of apoptosis, increasing proliferation and EMT, which result in local invasion and distant metastasis. In pancreatic cancer, the endoneurial macrophages secrete high levels of GDNF and its inhibition reduced perineural invasion in a mouse model [40,92]. GFLs enhanced the expression of integrins, the upregulation of matrix metalloproteinases (MMP-2, MMP-9), leading to pancreatic cancer cell motility and invasiveness into nerves, resulting from potential injury or inflammation [93,94]. In addition, ARTN has been reported to promote angiogenesis and metastasis through activation of the Twist-related protein (TWIST)1-vascular endothelial growth factor (VEGF)-A signaling in breast cancer [95]. GDNF is also able to increase VEGF-VEGFR interaction enhancing the migration of colon cancer cells [96]. In case of glioma or breast cancers, the GDNF-Ret signaling pathway can be blocked by GAS1 (growth arrest-specific 1) or by some Ret kinase inhibitors leading to the impairment of tumor growth [97,98]. GFLs are also linked with hepatocellular carcinoma (HCC), as high expression of GFRα3 or phosphorylation of Ret in HCC tissues and an increase of ARTN in the serum correlate with poor prognosis in HCC patients. ARTN, produced by tumor-inducible erythroblast-like cells, promotes HCC survival and invasion by inhibiting caspase activation and apoptosis initiation [99]. In neuroendocrine tumors, mutation of the RET proto-oncogene was identified as the causative gene for human papillary and medullary thyroid carcinoma, as well as for neuroendocrine small cell lung cancers, and early clinical trials of Ret inhibitors show encouraging results [100,101,102,103]. At last, GDNF and ARTN and their receptors GFRα1 and Ret stimulate radio- and chemoresistance via autophagy, mitogenesis and neutralizing apoptosis, suggesting that they may be potential therapeutic targets for overcoming chemoresistance in the case of osteosarcoma, neuroblastoma, mammary carcinoma and glioblastoma [91].

### 4.8. In Pain Sensitivity

Inflammation and injury lead to increased pain, resulting in the sensitization of sensory neurons to physical and chemical stimuli. There is accumulating evidence for a contribution of GFLs and GDNF-sensitive neurons to inflammatory pain in the context of skin, muscle, colon and skeletal pathologies [104,105]. GDNF, NRTN and ARTN have been shown to activate transient receptor potential vanilloid 1 (TRPV1) responses, leading to behavioral sensitivity to heat and cold [58,106]. In addition, ARTN potentiates this signaling in a subpopulation of sensory neurons via the coactivation of GFRα3 and TRPM8, a nociceptor involved in cold pain [107]. On the other side, GDNF/GFRα1 and NRTN/GFRα2 signaling pathways interfere with the inflammatory bone pain, through the activation and sensitization of nonpeptidergic neurons [104]. Finally, in irritable bowel syndrome (IBS), abdominal pain with motility issues are associated with increased levels of inflammatory cytokines and GFLs. These observations lead one to hypothesize that the use of Ret inhibitors may offer a novel therapeutic approach for the treatment of IBS [108]. Thus, GFLs and their receptors are potential targets for new inflammatory pain-controlling drugs [109,110].

## 5. Therapeutic Potential and Pharmaceutic Properties

GFLs have been proposed for potential cure of neurodegenerative diseases such as Alzheimer and Parkinson’s diseases, neuropathic pain, spinal cord injury, Huntington’s disease and ALS. However, GFLs failed to penetrate the blood-brain barrier which complicates their delivery and their clinical use for the therapies of nervous system disorders. The implementation of GFLs for treatment is difficult, as their biodistribution and bioavailability are low, and these molecules do not penetrate tissue barriers. To overcome these difficulties, small molecules with similar biological activity have been developed.

At the periphery, the exact mechanism of the GFL signaling pathway is not well characterized. In homeostatic conditions or in oncogenesis, the role of GFLs is complex and tissue dependent. Depending on the organs, GFRα1–4 can trigger the recruitment of RET, NCAM or TRPM8, showing that GFLs might exhibit pleiotropic effects in the body. Thus, the variability of receptors and co-receptors mediated by GFLs complicates the use of these molecules in the clinics, due to potential side effects [111]. On the other hand, GFLs can be used to target one specific tissue, as they have high affinity for extracellular matrix and cell surface heparan sulphate proteoglycans, limiting their diffusion in the organism. Thus, GFLs or the small molecules that mimic the effects of the different GFLs, could be proposed in addition to current therapies to treat the different pathologies evoked in this review (Table 1). But before this perspective, due to the complexity of GFL activities, challenges remain for further research to link experimental data with clinical significance.

## 6. Conclusions

The GFLs play a crucial role in the development of neurons and in epithelial-mesenchymal processes. In addition, GFLs are released consecutively to acute or chronic inflammation via epithelial-neuronal signaling, leading to the protection and the regeneration of the epithelial tissue. Following infection or injury, GFLs alert the body, through the activation of mechanisms of pain contributing to hypersensitivity and hyperalgesia. However, their roles are not limited to these functions, as they are also expressed by immune cells and regulate the secretion of pro-inflammatory cytokines. Their levels are upregulated in the serum of patients in several pathologies. It remains now to define exactly which pathways are involved and which effects are related to their release by immune cells. More studies are required to examine how their alterations could impact on disease processes.

## Figures and Tables

**Table 1:** Summary of the involvement of GFLs in different pathologies and models and their expression status or related mechanism of action. PI3K: phosphoinositide 3-kinase; Akt: protein kinase B; AhR: aryl hydrocarbon receptor; MMP: matrix metalloproteinase; TWIST1: twist-related protein 1; VEGFA: vascular endothelial growth factor A; VEGFR: vascular endothelial growth factor receptor; HCC: hepatocellular carcinoma; TRPV1: transient receptor potential cation channel subfamily V member 1; IBS: irritable bowel syndrome.

Pathology	GFL/Receptor Involved	Species	Expression Status or Related Mechanism of Action
Hirschsprung’s disease	RET	Human	Loss-of-function mutation
	GFRα1	Mouse	Reduction of expression
Crohn’s disease	GDNF	Human	Upregulation
Colitis	GDNF	Mouse	Expressed by enteric glial cells, regulates intestinal epithelial barrier integrity
Interstitial cystitis	GDNF	Human	Upregulation
Renal interstitial fibrosis	GDNF	Mouse	Expressed by adipose-derived mesenchymal stem cells, regulates macrophage activity
Diabetes	NRTN	Rat	Used for injections, prevents hyperglycemia
	GDNF	Mouse	Prevents enteric neuronal apoptosis via PI3K/Akt signaling activation
Diabetic retinopathy	GDNF and GFRα1	Rat’s cultured Müller cells	Expressed under high glucose conditions, protecting role
Asthma	GDNF	Guinea pig	Allergen sensitization induces its expression in airway mucosa and tracheal epithelium
	NRTN	Mouse	Inactivation of the molecule increases inflammation and airway remodelling markers
Eosinophilic chronic rhinosinusitis	GFRα2 and NCAM	Human	Downregulation in nasal polyps
Grass-pollen allergy	GFRα1–4, GDNF and NCAM	Human	Downregulation in nasal samples from patients under allergen-specific immunotherapy
*Streptococcus pneumoniae* infection	GDNF	Mouse	Downregulation in the olfactory bulb
Psoriasis	NRTN	Human	Upregulation in the skin
	NRTN and GFRα2	Mouse	Blocking the pathway reduces nonpeptidergic nerve density
Atopic dermatitis	ARTN	Human	Upregulation in epidermis via activation of AhR
			Accumulates in dermal fibroblasts and induces epidermal hyper-innervation
Mood disorder	GDNF and ARTN	Human	Downregulation in blood
Cancer	All GFL’s	Human	Upregulation in a variety of cancer cells of epithelial origin, associated with malignant progression and poor prognosis
	GDNF, ARTN, GFRα1 and RET		Stimulates radio and chemoresistance via autophagy, mitogenesis and neutralizing apoptosis
Pancreatic cancer	GDNF	Mouse	Inhibition of its expression from endoneurial macrophages reduces perineural invasion
	All GFL’s	Human	Enhances integrin expression and the upregulation of MMP
Breast cancer	ARTN	Human cell lines	Promotes angiogenesis and metastasis via TWIST1-VEGF-A signaling
Colon cancer	GDNF	Human	Increases cancer cell migration via VEGF-VEGFR interaction
Breast cancer and glioma	GDNF and RET	Human cell lines	Blocking of the pathway leads to the impairment of tumor growth
Hepatocellular carcinoma	GFRα3, RET and ARTN	Human	Upregulation, correlates with poor prognosis
	ARTN	Mouse	Expressed by tumor-inducible erythroblast-like cells, promotes HCC survival and invasion
Neuroendocrine tumors	RET	Human	Loss- of-function mutation leads to papillary, medullary thyroid carcinoma and neuroendocrine small cell lung cancers
Pain sensitivity	GDNF, NRTN, ARTN and GFRa3	Mouse	Sensitivity to heat and cold via TRPV1 signaling
Inflammatory bone pain	GDNF, NRTN, GFRα1–2	Rat	Via activation and sensitization of nonpeptidergic neurons
Abdominal pain (IBS)	RET	Rat	Inhibition attenuates the number of abdominal contractions via visceral nociception
